# Facile Preparation of Flexible Phenolic-Silicone Aerogels with Good Thermal Stability and Fire Resistance

**DOI:** 10.3390/molecules30030464

**Published:** 2025-01-21

**Authors:** Zengyue Su, Zhenrong Zheng, Xiaobiao Zuo, Lijuan Luo, Yaxin Guo

**Affiliations:** 1 College of Textile and Engineering, TianGong University, Tianjin 300387, China; 2 National Engineering Research Center of Functional Carbon Fiber Composition Materials, Beijing 100076, China; 3 Aerospace Research Institute of Materials and Processing Technology, Beijing 100076, China; 4 College of Fine Arts, Linyi University, Linyi 276000, China

**Keywords:** hybrid aerogel, phenolic resin, organosilicone, flexibility, ambient pressure drying

## Abstract

A huge challenge is how to prepare flexible silicone aerogel materials with good flame retardancy, thermal stability, and hydrophobic properties. In this paper, resorcinol–formaldehyde was introduced into the silicone network composed of methyltrimethoxysilane (MTMS), phenyltriethoxysilane (PTES), and dimethyldimethoxysilane (DMDMS). Flexible hybrid aerogels with excellent thermal insulation, flame retardant, and hydrophobic properties were prepared by the sol–gel method and ambient pressure drying (APD), and the preparation process does not require long-term solvent exchange, only about 3 h of soaking and washing of the wet gel. The results show that the prepared phenolic-silicone aerogel has low density (0.093 g/cm^3^), low thermal conductivity (0.041 W/m·K), high flexibility, and compression fatigue resistance. The phenolic microspheres are bonded to the silicone skeleton to maintain the original flexibility. After 50% compression deformation, it returns to the original size normally, and there is no significant change in the stress of the sample after 50 compression cycles. Compared with pure silicone aerogels, the hybrid aerogels doped with phenolic have better char yield (65.28%) and higher decomposition temperature (609 °C). The hybrid aerogel sample has good flame-retardant properties, which can withstand alcohol lamp burning without being ignited. The micron-sized phenolic beads give the hybrid aerogels better hydrophobic properties, showing a higher static water contact angle (152°). The excellent thermal and mechanical properties mean that the hybrid aerogels prepared in this paper have good application prospects for aerospace, outdoor equipment, and other fields.

## 1. Introduction

Aerogels are known for their excellent thermal insulation properties due to their low density and high specific surface area [1,2,3]. Currently, various types of aerogels have been developed, such as silica aerogels [4,5,6], carbon aerogels [7,8], phenolic aerogel [9,10], and some composite aerogels [11,12,13]. Among them, silica aerogels have been widely used as thermal insulation materials [14,15]. However, the brittleness limits their application in fields like artificial intelligence and aerospace. Therefore, it is crucial to develop aerogel materials with flexibility and low thermal conductivity [16,17,18] through ambient pressure drying methods.

Silica is a material known for its flame retardant and thermal insulation properties [19], and fumed silica particles are commonly used as inorganic fillers in flame-retardant coatings. Consequently, silica aerogels [20,21], prepared using silanes as raw materials, are often employed for flame retardancy and thermal insulation. The preparation of traditional silica aerogels requires the use of supercritical equipment, which is expensive and difficult to obtain [22,23]. The prepared hard silicon aerogels are brittle and restrict use. When methyl-containing silicon sources, such as methyltriethoxysilane (MTES) [24,25] and methyltrimethoxysilane (MTMS) [26], are used to prepare silica aerogels, the methyl groups do not participate in the dehydration–condensation process. This gives the otherwise rigid chains a degree of flexibility [27], resulting in the production of organosilicone aerogels with some flexibility [28]. However, the introduction of methyl groups into the inorganic silica system introduces organic components, which compromises the original flame retardancy and thermal insulation properties. Therefore, the introduction of phenyl groups [29] should be considered to enhance the thermal stability of organosilicone aerogels.

Phenolic resin is one of the most extensively studied resins, often used as a thermal protection material [30,31]. However, phenolic aerogels obtained through the sol–gel method are typically hard and brittle [9,32]. To address this, many researchers have developed various types of phenolic-silicone aerogels [17,33,34,35] by hybridizing phenolic resin with organosilicone. Most of these preparation processes involve the use of pre-made water-soluble phenolic resins and the crosslinking agent hexamethylenetetramine (HMTA) [36]. For example, He et al. [37] used HMTA as a curing agent to crosslink short-chain phenolic molecules with long-chain silicone to obtain a long-distance cross-linked hybrid molecule, thereby preparing a flexible phenolic-silicone aerogel. It has good reversible compressibility, excellent thermal insulation, and hydrophobicity. A significant drawback of pre-made water-soluble phenolic resins is their difficulty in storage; they tend to cure even at low temperatures, making them challenging to use and problematic for industrial production. Additionally, HMTA as a curing agent is classified as an explosive precursor, posing safety risks.

The phenolic [38] component is an important part of improving the thermal stability and carbon residue of aerogels. In the synthesis process, how to avoid the use of water-soluble phenolic resin and explosive precursor HMTA, which are difficult to preserve, and introduce phenolic components into the silicon system, is a problem. Therefore, it is essential to develop a method for preparing flexible [16,39] phenolic-silicone aerogel starting from silane, phenol, and formaldehyde.

In this paper, urea, resorcinol, and formaldehyde were mixed in water to obtain a neutral aqueous solution. In the process of water bath heating, the resorcinol and formaldehyde were pre-polymerized in a neutral and high-temperature aqueous solution. After mixing with silica sol, the hybrid aerogel sample with high thermal stability was prepared. The phenolic component in the form of microspheres attached to the silicone skeleton. The hybrid aerogel not only has the flexibility brought by the long-chain silicon molecular skeleton but also obtains thermal stability and high carbon residue rate of the phenolic component and has both hydrophobic and flame-retardant properties. The by-product urea–formaldehyde resin prepolymer is decomposed when it enters the acidic silica sol, which does not affect the aerogel synthesis.

## 2. Results

### 2.1. Fabrication of Sample S1–S5

**Figure 1:**
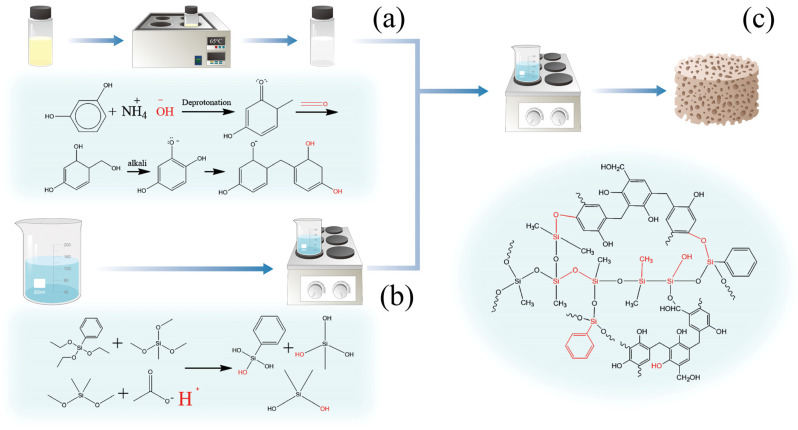
(**a**) Polymerization reaction of phenol and aldehyde in alkaline environment. (**b**) Hydrolysis of siloxane in acidic environment. (**c**) Drying the sol after fully stirring.

**Figure 2:**
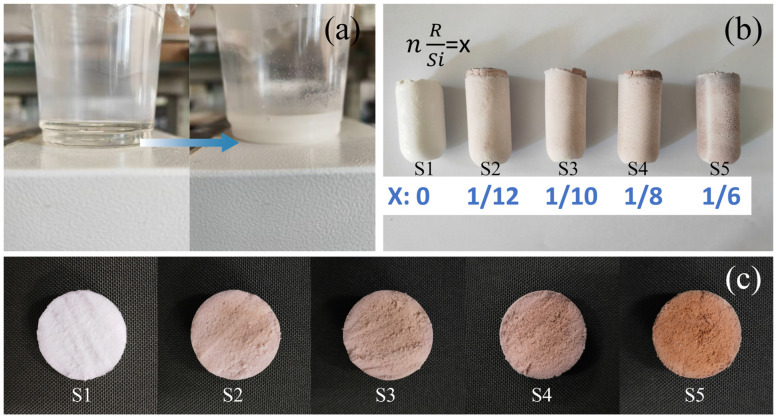
(**a**) Changes from phenolic aqueous solution to emulsion. (**b**) Macroscopic morphology of aerogels with different molar additions. (**c**) The cross sections of samples S1–S5.

To enhance the thermal stability and carbon yield of flexible silicone aerogels, phenolic resin was incorporated into the silicone network. The synthesis process and the molecular bonding demonstration are shown in Figure 1. The brittleness of the phenolic structure was mitigated by the introduction of flexible silicone chains. Urea was added to a resorcinol–formaldehyde solution, and with the pH adjusted to 7, the mixture was heated in a water bath within a glass container. During the reaction, urea and formaldehyde formed hydroxymethylurea and dihydroxymethylurea, while resorcinol and formaldehyde produced monomeric hydroxymethylphenol and polyhydroxymethylphenol. The solution in the vial changes from a transparent to white emulsion, as shown in Figure 2a. These intermediates subsequently underwent polycondensation, resulting in a macromolecular resin. This turbid suspension is then combined with the hydrolyzed silane solution, and additional urea is introduced to adjust the pH, maintaining it near neutral. A neutral pH favors the slow polycondensation of silicone, preventing excessive polymerization and the formation of coarse structures. Dehydration condensation occurs between the phenolic hydroxyl group contained in the phenolic resin and the silicon hydroxyl group produced by the hydrolysis of siloxane. The phenolic component is grafted onto the silicone molecular chain and cured at high temperature to achieve the adhesion of phenolic resin.

The uniformly dispersed phenol–urea–formaldehyde polycondensate also reacts with the silanol groups on the silicone’s long chains, forming phenol–urea–aldehyde short-chain molecules attached to the silicone backbone. This allows phenol–urea–aldehyde components to be incorporated without compromising the flexibility of the silicone chains, thereby enhancing the thermal properties of the aerogel. The macroscopic morphology of the aerogel sample is shown in Figure 2b. Let the molar ratio of phenolic aldehyde to silicon element be x. The samples are arranged from left to right, and the content of phenolic aldehyde is increasing. In order to make it more convenient to express, the samples are named S1–S5 according to the trend of increasing phenolic aldehyde content. The cross-sectional images of samples S1–S5 are shown in Figure 2c. From S2–S5, with the increase in phenolic aldehyde content, the color of the cross sections tends to be reddish brown. From S2–S5, the brittleness of the sample increases, and the cutting feel is closer to the foam.

**Figure 3:**
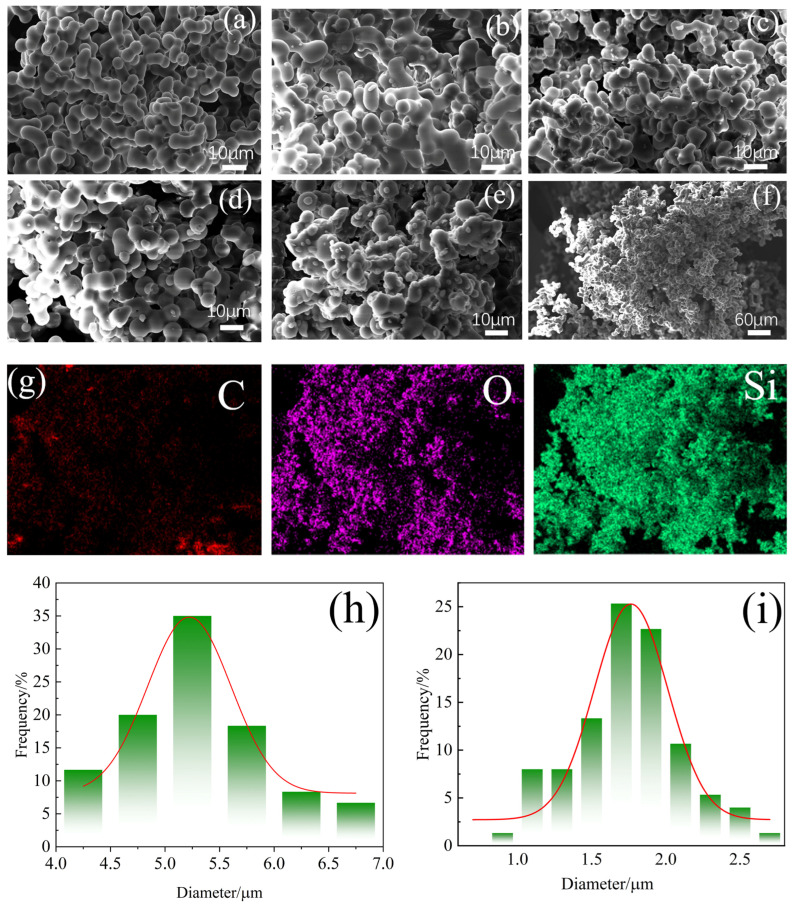
(**a**–**e**) SEM of S1-S5. (**f**,**g**) SEM and EDS of sample S3. (**h**,**i**) The particle size of silicon microspheres and phenolic microspheres.

The aerogel sample obtained has a similar basic structure, that is, the silicone network, as shown in Figure 3a. MTMS and DMDMS are connected to form a long silicone chain, and PTES forms a coating layer on the outside of the silicone chain, enhancing the connectivity of the silicone chain. The coated PTES will destroy the mechanical properties of the aerogel to some extent, but the introduction of phenyl will enhance the thermal stability of the aerogel.

Figure 3a is the SEM image of the silicone aerogel S1 sample with a phenolic addition of 0 mol, and Figure 3b–e show the SEM images of the S2–S5 samples with different phenolic additions (Mag = 1000X). Many small phenolic resin microspheres formed by the polymerization of phenolic resin appeared outside the originally smooth silicon spheres and adhered to the silicone network through chemical bonding. The SEM image of the sample S3 (Mag = 300X) and the corresponding EDS scanning image are shown in Figure 3f–g. The EDS image shows the presence, and the distribution of C, O, and Si elements can confirm that there is no phase separation between phenolic and silicone. Figure 3a,e was imported into ImageJ software (2.1.4.7), thirty microspheres were selected to record their diameter data, and placed in Origin software (2019b) to find the distribution law of their particle size. Using Figure 3a,e as examples, the particle size data of silicon spheres and phenolic microspheres were extracted and the particle size diagrams were drawn, as shown in Figure 3h–i. It can be seen that the particle size distribution of silicon spheres was mostly 5~5.5 μm, and that of phenolic microspheres was mostly 1.5~2 μm.

### 2.2. Chemical Structure Characterization

**Figure 4:**
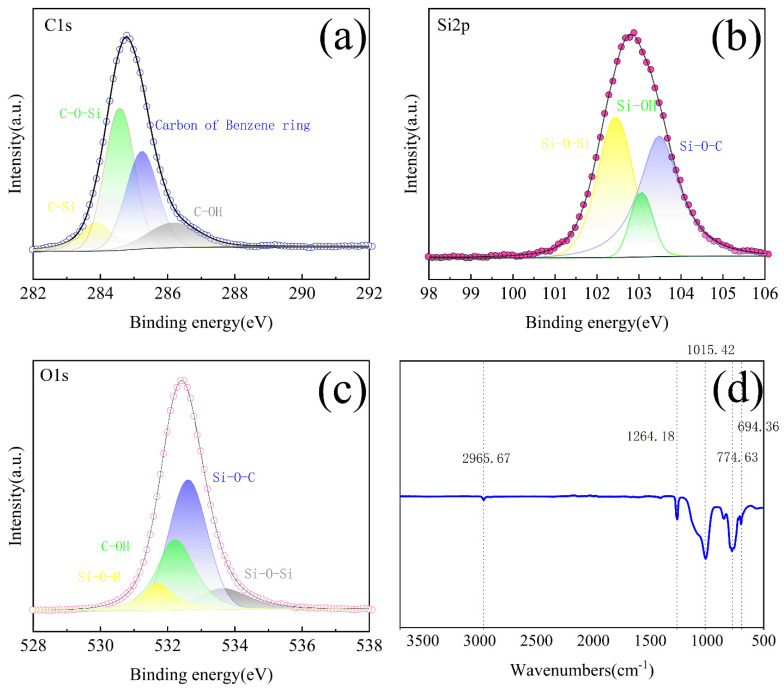
(**a**−**c**) C1s, Si2p, and O high-resolution XPS spectra of S3. (**d**) FTIR spectra of S3.

The chemical structure of the aerogel sample S3 was characterized by FTIR spectroscopy and XPS. As shown in the Figure 4, the absorption peak observed at 1015 cm^−1^ and 774 cm^−1^ is caused by the vibration of the Si-O-Si bond, the absorption peak at 1264 cm^−1^ is caused by the deformation vibration of -CH_3_, and the peak at 2965 cm^−1^ is caused by the antisymmetric contraction vibration of CH_3_. The peak at 694 cm^−1^ indicates the benzene mono-substituted structure of Si-C_6_H_5_ [29].

The C1s peaks at 283.8, 284.2, 284.6, and 285.4 eV correspond to C-Si, C-O-Si, the carbon atom of the benzene ring, and C-OH, respectively [34]. The Si2p peaks at 101.9, 102.4, 103, and 103.8 eV correspond to the binding energies of Si-O-Si, Si-OH, Si-O-C, and Si-C bonds, respectively. The peaks at 531, 531.5, 532.2, and 532.9 eV correspond to Si-O-H, C-O-H, Si-O-C, and Si-O-Si these chemical structures [37], where C-O-H and Si-O-C represent the presence of phenolics and the connection with the silicon component.

### 2.3. Thermal Performance 

**Figure 5:**
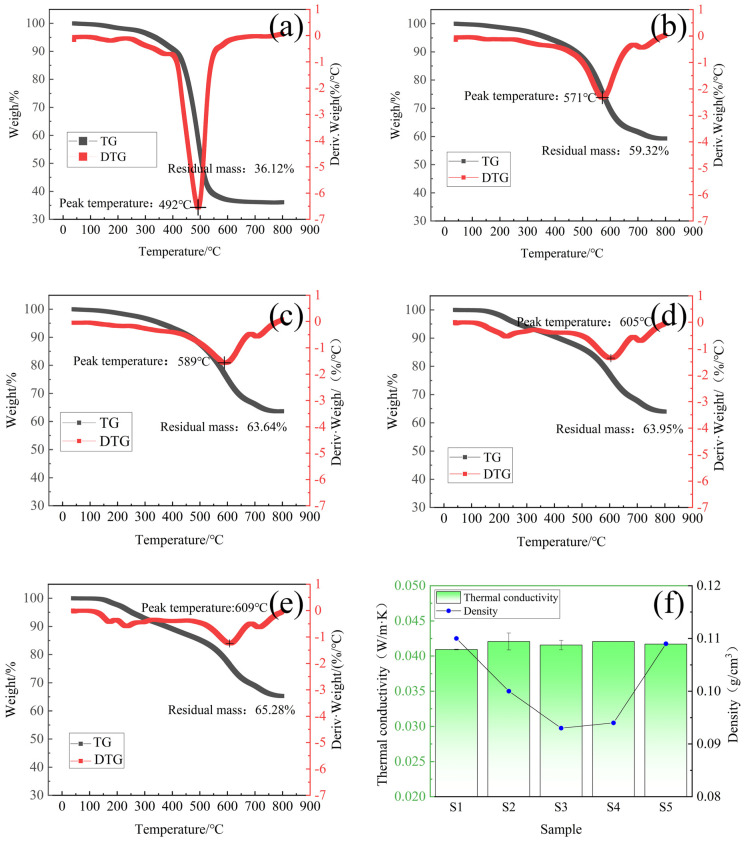
(**a**–**e**) TGA and DTGA curves of S1–S5 under nitrogen atmosphere. (**f**) Thermal conductivity and bulk density of sample S1–S5.

The TGA curves of S1–S5 are shown in Figure 5a–e. For phenolic-silicone aerogel, the first stage of weight loss comes from the release of water and the further condensation of phenolic resin. This reaction occurs in the early stage of weight loss, and the weight loss rate is about 10% before the temperature is 400 °C. Starting from 400 °C, the pyrolysis rate begins to increase, and silicone and phenolic resin are separated and pyrolyzed, reaching the peak value at about 571 °C to 609 °C. At this time, the methylene bridge of phenolic resin is broken.

The carbon yield and peak pyrolysis temperature of hybrid aerogel are higher than those of silicone aerogel. Compared with other reported aerogels (52% [30], 61.52% [31]), hybrid aerogel has obvious advantages in carbon yield and deposition temperature, which proves that the material has good thermal stability.

The thermal conductivity of silicone aerogel S1 measured at room temperature of 25 °C was 0.0409 W/(m·K), and the thermal conductivity of phenolic-silicone aerogel was slightly higher than that of silicone aerogel, but the overall thermal conductivity was stable between 0.041 and 0.043 W/(m·K), and the S3 sample had the lowest density (0.093 g/cm^3^) according to the bulk density data shown in Figure 5f. It was preliminary confirmed that the phenolic-silicon molar ratio of 1:10 had the best effect, and the prepared sample had the advantages of low density, low thermal conductivity, and a high carbon residue ratio.

**Figure 6:**
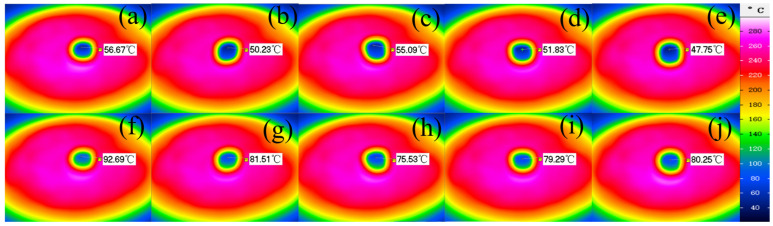
(**a**–**e**) The infrared temperature diagram of samples S1–S5 heated in a heating plate for 10 s. (**f**–**j**) The infrared temperature diagram of samples S1–S5 heated in a heating plate for 300 s.

In order to further evaluate the thermal insulation performance of the aerogel sample, the aerogel sample with a thickness of 10 mm was placed on a heating plate with a temperature of about 280 °C, and the back temperature was recorded with an infrared imager. As shown in Figure 6f, the temperature of silicone aerogels rose to 92.69 °C after 5 min, showing the worst performance among the 5 samples. The back temperature of phenolic-silicone aerogels stabilized at about 80 °C after heating for 5 min, as shown in Figure 6g–j. Compared with pure silicone aerogel, the back temperature of silicone phenolic hybrid aerogel is lower after heating the heating plate for 5 min, which proves that the introduction of phenolic resin has a certain enhancement on the thermal insulation performance of aerogel. Figure 6h shows that the back temperature of the S3 sample is about 75 °C. The reason why the S3 sample can achieve a lower back temperature is we speculate that in addition to its lower density, the phenolic component in the carbonized carbon layer with lower thermal conductivity is a more important factor. Compared with S5, its phenolic component content is lower, and the heat generated during carbonization is relatively small, so the back temperature is also lower than that of the S5 sample.

**Figure 7:**
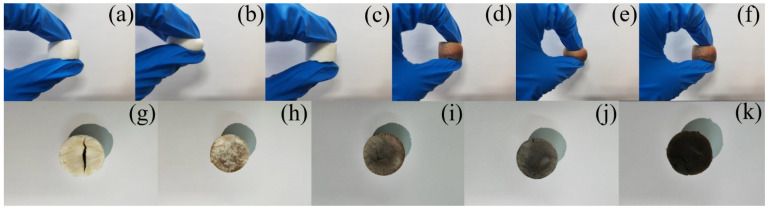
(**a**–**c**) Flexibility test of S1 samples after 280 °C heating plate test. (**d**–**f**) Flexibility test of S3 samples after 280 °C heating plate test. (**g**–**k**) the macroscopic morphology of sample S1–S5 after heating plate test at 600 °C for 30 s.

There was no cracking on the surface of the silicone aerogel and no change in color. As can be seen from Figure 7a–f, at a temperature of about 280 °C, the aerogel maintained its flexibility and could recover itself immediately after being pinched by hand. The contact surface of the phenolic-silicone aerogel with the heating plate became black after being ablated at 280 °C for 5 min, and the surface did not crack but also maintained its flexibility and self-recovery ability after external deformation.

In order to explore the limit temperature of the aerogel, the temperature of the heating plate was raised to 600 °C, and the aerogel was placed on the heating plate for about 30 s, and a large amount of white smoke was gradually released. Cracks appeared on the surface of the silicone aerogel, and the aerogel was removed from the heating plate, and a large amount of smoke was released without stopping the carbonization process. Phenolic-silicone aerogel, after heating for a period of time, also released a lot of smoke, but the surface did not crack. This is due to the introduction of the phenolic carbon chain structure, so that the aerogel structure is not so fragile and will not actively crack after heating. The thermal stability of the aerogel was improved. The phenolic-silicone aerogel was removed from the heating plate, and the carbonization process was continuous, and after the carbonization process was completed, it became a fragile block as shown in Figure 7g–k.

### 2.4. Flame Resistance

**Figure 8:**
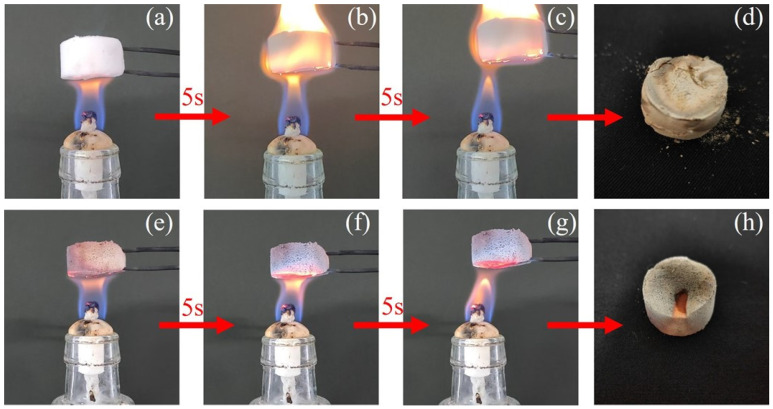
(**a**–**c**) Photographs of S1 aerogels burned with alcohol lamp. (**d**) Photograph of sample S1 after burning. (**e**–**g**) Photographs of S3 aerogels burned with alcohol lamp. (**h**) Photograph of sample S3 after burning.

The flame-retardant properties of silicone aerogel S1 and phenolic-silicone aerogel S3 were verified by alcohol lamp. As shown in Figure 8a–c, the pure silicone aerogel S1 was quickly ignited after contacting the flame, and the entire aerogel block was wrapped by the flame. After the flame was removed, the aerogel still burned violently. The aerogel S3 did not burn after contacting the flame, and the heating of 10 s did not ignite it as shown in Figure 8e–g, showing good flame retardancy.

The bottom of S3 phenolic-silicone aerogel became red during the ablation process of the fire filling. From the comparison of Figure 8d,h, it can be seen that the flame retardancy of the S3 sample may be due to the fact that the phenolic component is not flammable and easy to form a stable carbon layer after combustion, and the carbon layer has good shape retention. After combustion, the S3 sample still maintains a block structure and does not collapse. The carbonized aerogel maintains a complete shape and is suitable for one-time high-temperature thermal insulation protection.

### 2.5. Aperture and Contact Angle

**Figure 9:**
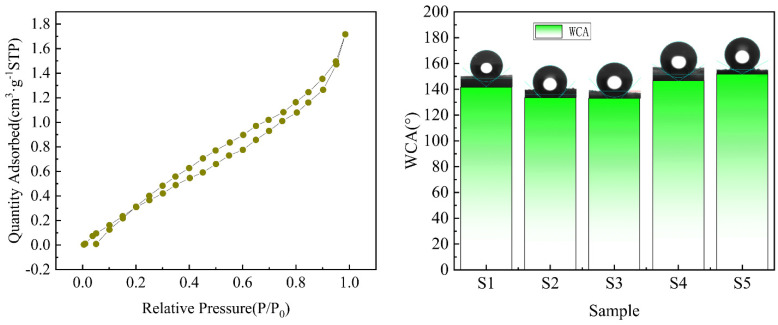
Nitrogen adsorption–desorption hysteresis curve and static contact angle.

The N_2_ adsorption and desorption isotherm of the phenolic-silicone aerogel S3 sample is shown in Figure 9. According to the IUPAC classification, the adsorption and desorption isotherm of the sample belong to the type II adsorption isotherm, and the adsorption hysteresis is obvious, indicating the adsorption of N_2_ on the mesoporous adsorbent and the single-layer adsorption of capillary condensation. Without the appearance of an adsorption platform, the pore structure may not be regular, indicating the existence of mesoporous and macro porous structures in the internal structure of aerogel.

The static contact angle of the pure silicone aerogel S1 is 141.6°, which is hydrophobic but does not reach the level of superhydrophobicity. This is because DMDMS and MTMS are the basic skeleton of silicone aerogels, and there is a lot of -CH_3_ on the surface, which makes pure silicone aerogels show good hydrophobicity. After the introduction of phenolic aldehyde, the contact angles of S2 and S3 hybrid samples decreased to a certain extent due to the hydrophilicity of phenolic aldehyde with partial OH. With the increase in phenolic aldehyde content, the number of phenolic aerogel particles (1.5~2 μm) attached to the silicone skeleton increased significantly, almost completely covering the silicone skeleton, resulting in a structure similar to the lotus leaf structure. This structure further improved the hydrophobicity of the hybrid aerogel, and the hydrophobic angle was up to 152°, so that the sample had superhydrophobic properties.

### 2.6. Mechanical Property

**Figure 10:**
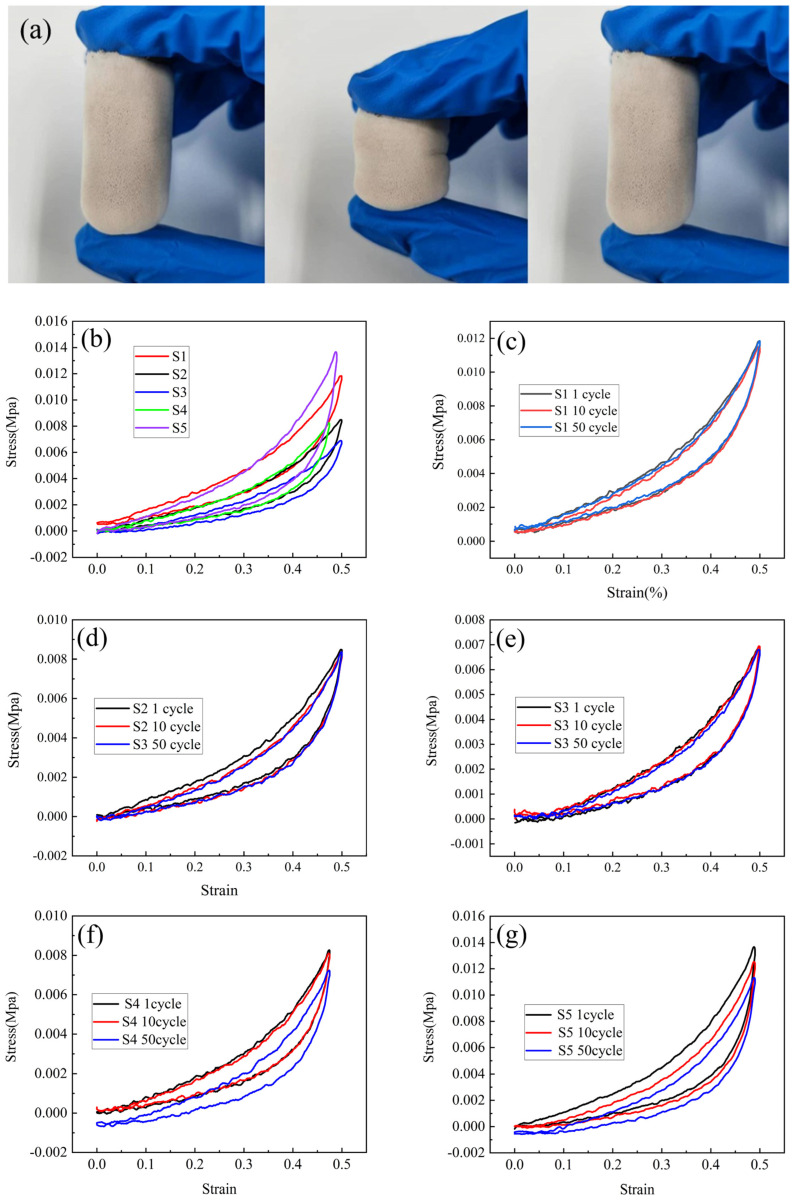
(**a**) Sample S3 finger compression rebound test. (**b**) Stress comparison of samples S1–S5 at 50% strain degree. (**c**–**g**) Stress–strain comparison of samples S1–S5 at 1, 10, and 50 cycles.

The combination of silicone network molecular structure and gel pore structure gives the hybrid aerogel good flexibility, which can withstand the corresponding external extrusion and produce elastic deformation and recover after the external force is removed. As shown in Figure 10a, the macrostructure of the aerogel was not destroyed after the finger was compressed with an uneven external force, and the shape recovered after the external force was removed.

The compression–decompression mechanical properties of aerogel samples were tested, and the flexibility of each sample was compared. The compressive stress–strain (σ-ε) curve is shown in Figure 10b–g. The obvious hysteresis phenomenon on the stress–strain curve of the aerogel may be caused by the viscoelasticity of the silicone skeleton network structure.

As shown in Figure 10b, under 50% compressive strain, the stress of silicone aerogel S1 is about 0.012 MPa, which has good flexibility and certain mechanical properties. It can be seen from Figure 10b that from S1 to S5, the peak stress of the sample decreases first and then increases. It may be due to the introduction of phenolic resin. The filling of the phenolic resin microspheres reduced the deformation space of the aerogel molecular chain. The maximum compressive stress of sample S4 is higher than that of sample S3, and the compressive stress of sample S5 reaches the peak value of 0.013 MPa.

In order to further analyze the compressive resilience of aerogels, the σ-ε curves of aerogels were analyzed after 50 cycles of 50% compressive strain. As shown in Figure 10c–e, after 50 cycles of compression–decompression, the stress slope of aerogels did not change significantly. It is attributed to the high flexibility and deformability of the CH_3_ group outside the aerogel, which can expand the contact area, release the stress, and reduce the stress of the Si-O-Si skeleton. The aerogel shows good softness, and its compressive fatigue resistance and durability are good. It can be seen from Figure 10f–g that as the number of compression cycles increases, the stress of samples S4 and S5 tends to decrease, after 50 compression cycles, the stresses were reduced to 0.007 and 0.011 MPa, respectively. This indicates that the excess introduction of phenolic resin leads to the deterioration of compressive fatigue resistance of aerogels.

An appropriate amount of phenolic resin can maintain flexibility and fatigue resistance. As shown in Figure 10e, after 50 compression cycles, the stress of the S3 sample did not decrease significantly, and its thermal performance was only slightly worse than that of S5 sample, and the carbon residue rate was 63.64%. Combining the two evaluation indexes, the S3 sample with a molar ratio of resorcinol to silicon of 1:10 has the best performance. S3 sample balance flexibility and good thermal properties should be used as the preferred material for thermal insulation applications.

## 3. Materials and Methods

### 3.1. Materials and Chemicals

Methyltrimethoxysilane (MTMS), dimethyldimethoxysilane (DMDMS), phenyltriethoxysilane (PTES), cetyltrimethyl ammonium chloride (CTAC), and urea were purchased from Tianjin Medin Technology Co., Ltd., Tianjin, China. Resorcinol was purchased from Tianjin Yifang Technology Co., Ltd., Tianjin, China. Formaldehyde solution was obtained from Kemat (Tianjin) Chemical Technology Co., Ltd., Tianjin, China. Acetic acid was purchased from Tianjin Fengchuan Chemical Reagent Technology Co., Ltd., Tianjin, China.

### 3.2. Fabrication of Hybrid Aerogel

First, resorcinol and formaldehyde were added to a sealed vial containing 0.1 g urea and 4 mL water at a molar ratio of 1:2. Then the vial was heated in a water bath, and the temperature gradually increased from 25 °C to 85 °C. In this process, the solution became a white emulsion. Next, 1.2 g of CTAC was added to a beaker with 30 μL of acetic acid and 15 mL of deionized water, and the mixture was magnetically stirred until the CTAC was completely dissolved. Then MTMS 3 mL, DMDMS 2 mL, and PTES 0.6 mL were added. After stirring for 10 min, 5 g of urea and phenolic urea–formaldehyde prepolymer emulsion were added to the vial. Then, the mixture was stirred for 1 h, then transferred to a sealed vial, placed in an oven at 85 °C for 6 h, and aged in a 60 °C incubator for 18 h.

The extracted wet gel was washed twice with deionized water, soaked in a methanol solution for 4 h, and washed again twice with methanol to remove impurities. The sample was then dried at room temperature and pressurized. Five different aerogel samples were prepared based on varying amounts of resorcinol and were designated as S1–S5. The molar quantity of phenolic was 0, 0.003, 0.0036, 0.0045, and 0.006 mol. The molar ratios of silicon to phenolics in samples S2–S4 were 12, 10, 8, and 6.

### 3.3. Characterization

The micromorphology of the phenolic-silicone aerogel was observed and characterized using a thermal field-emission scanning electron microscope (SEM, GeminiSEM 500, ZEISS, Cambridge, UK). The functional groups and bonding changes within the aerogel were analyzed by Fourier transform infrared spectroscopy (FTIR, Nicolet iS50, Thermo Fisher Scientific, Waltham, MA, USA) over the wavenumber range of 4000–400 cm^−1^, as well as by X-ray photoelectron spectroscopy (XPS, NEXSA, Thermo Fisher Scientific, Waltham, MA, USA). The thermal stability of the aerogel samples was assessed using a thermogravimetric analyzer (TG 209 F3 Tarsus, NETZSCH-Gerätebau GmbH, Selb, Germany) by heating the samples from 25 °C to 800 °C at a rate of 10 °C/min in a nitrogen atmosphere. A fully automated surface area and porosity analyzer (NOVA 4200e, Anton Paar Quanta, FL, USA) was used to determine the specific surface area and mesoporous pore distribution of the samples. The thermal conductivity of the samples was measured by a thermal constant analyzer (TPS-2500s Hot Disk, Hot Disk AB, Gothenburg, Sweden) at room temperature at 25 °C. The flame retardancy of the samples was tested using an alcohol lamp. Compression–decompression tests were conducted on a universal testing machine (Instron 5969, Instron, Boston, MA, USA) with sample dimensions of 10 mm × 20 mm and a compression rate of 5% deformation per second. The mass of each sample was measured using an electronic balance, and the radius and length were measured with a vernier caliper to calculate density by dividing mass by volume. The thermal insulation performance of the samples was evaluated on a universal heating plate, with real-time temperature monitoring performed using a thermal imaging camera (Infra Tec, Infra, Dresden, Germany). The hydrophobic properties of the samples were tested using a static water contact angle tester (OCA 15pro, DataPhysics Instruments, Stuttgart, Germany).

## 4. Conclusions

In summary, phenolic-silicone aerogel aerogels were prepared by the sol–gel method with MTMS, DMDMS, and PTES siloxane as precursors and phenolic prepolymer as the modifier. The phenolic component was introduced as a role in heat insulation, increasing carbon residue and thermal stability. The results show that the introduction of the phenolic component changes the microstructure of the silicone aerogel, and the phenolic component is bound to the silicone skeleton in the form of microspheres. Compared with the silicone aerogel without phenolic component, the addition of phenolic resin with a molar ratio of silicon to phenolic resin of 10:1 can increase the carbon residue rate of the aerogel from 36.12% to 63.64%, and the peak decomposition temperature increased from 492.23 °C to 588.69 °C. The addition of phenolic aldehyde makes the aerogel obtain obvious flame-retardant ability, and its rapid charring performance provides a basis for the flame retardant of the aerogel. The carbonized aerogel remains intact without collapse and damage. The aerogel also has good hydrophobic properties. It has been proven that the hybrid aerogel material has a good application prospect in the fields of fire prevention and heat insulation.

## Data Availability

Data will be made available on request.
